# Shining a light on postnatal perineal granulation tissue

**DOI:** 10.1002/ijgo.14297

**Published:** 2022-06-25

**Authors:** Caroline Brophy, Gillian A. Corbett, Laoise O′Brien

**Affiliations:** ^1^ Postnatal Morbidity Service (POPPY Clinic), National Maternity Hospital Dublin Ireland; ^2^ Department of Midwifery, National Maternity Hospital Dublin Ireland; ^3^ Department of Obstetrics and Gynaecology, National Maternity Hospital Dublin Ireland; ^4^ UCD School of Medicine University College Dublin Dublin Ireland

**Keywords:** delivery, granulation tissue, obstetric labor complications, parturition, perineum, postpartum period, pregnancy

## Abstract

Perineal granulation tissue affects 2.7% of vaginal deliveries. Associated with nulliparity and instrumental delivery, it causes pain and delays recovery for new mothers.

The postnatal period is a significant and vulnerable time when complications can delay recovery and increase distress for new mothers.^1^, ^2^ Postnatal perineal granulation tissue (PPGT) is often observed by healthcare providers in both primary care and maternity settings, and can cause significant morbidity. The National Maternity Hospital has run a dedicated postnatal morbidity service for almost 10 years. The POPPY Clinic is a unique clinic caring for postnatal women experiencing complications such as PPGT during recovery after delivery. In order to expedite recovery, the clinic practices active management of PPGT, offering women conservative, medical (topical silver nitrate), or surgical (resection under local or general anesthesia) treatment for PPGT.

Despite being commonly observed, there is a distinct lack of evidence on PPGT.^3^ The present study aimed to establish the incidence of PPGT along with risk factors, symptomatology and treatment efficacy. Ethical approval was granted for the granulation tissue study by the center's institutional review board on December 13, 2021 (reference number EC31.2021). Informed consent was not obtained because this was a retrospective clinical audit.

A retrospective review of activity at the POPPY Clinic was performed over a 3‐month period (June–August 2021). Cases of PPGT were identified opportunistically by POPPY clinicians or the Advanced Midwife Specialist at scheduled hospital‐based follow up, emergent re‐presentation, or referral from the primary care setting.

The incidence of PPGT among women with vaginal delivery was 2.7% (31/1162). Nulliparous women had significantly higher incidence of PPGT (6.1%, 28/462 nulliparas versus 0.43%, 3/700 multiparas (odds ratio [OR] 15.0, 95% confidence interval [CI] 4.53–49.60). Amongst nulliparas (Table 1), PPGT was significantly more likely with instrumental deliveries (OR 2.90, 95% CI 1.32–6.20), particularly with sequential instrumental deliveries (OR 5.70, 95% CI 1.43–22.67). Most affected women had a body mass index (calculated as weight in kilograms divided by the square of height in meters) below 30 (90.3%, 28/31).

**TABLE 1:** Incidence of postnatal perineal granulation tissue among nulliparous women with vaginal delivery (*n* = 462)

Mode of delivery	Incidence of PGT in nulliparas (6.1%)	Number of PGT/Total number of vaginal deliveries (28/462)	OR^a^ (95% CI) compared with SVD
Spontaneous vaginal delivery	3.9%	12/308	
Assisted vaginal birth	10.4%	16/154	2.90^a^ (1.32–6.20)*
Kiwi forceps delivery with RMLE	7.4%	7/94	2.0^a^ (0.76–5.20)
Forceps delivery with RMLE	13.6%	6/44	3.90^a^, ^b^ (1.38–10.98)*
Sequential instrumental delivery (Kiwi forceps) with RMLE	18.8%	3/16	5.70^a^ (1.43–22.67)*

Abbreviations: CI, confidence interval; OR, odds ratio; PGT, perineal granulation tissue; RMLE, right mediolateral episiotomy; SVD, spontaneous vaginal delivery.

^a^ Odds ratio.

^b^ Relative risk.

* Significant at *P* < 0.05.

The most common issues for women with PPGT were perineal pain (96.8%, 30/31) and fear of resuming sexual activity (77.4%, 24/31). Anatomical sites of PPGT included the vaginal fourchette (35.4%,11/31), the vaginal mucosa (32.3%,10/31), and the external aspect of the episiotomy scar (32.3%, 10/31).

All PPGT recognized before six postnatal weeks were managed conservatively. Conservative management was 40% effective (7/20), requiring subsequent medical (50%, 10/20) or surgical (15%, 3/20) treatment. Medical management, offered between 6 and 12 weeks postnatally, was effective in 100% of women. Surgical management, reserved for persistent granulation tissue from 12–44 weeks postnatally, was 100% effective with no instances of recurrence.

The incidence of PPGT was 2.7%, but ranged up to 6.1% in nulliparas and was more common with instrumental delivery. Given its prevalence, it is incumbent on maternity services to provide dedicated specialized care for women experiencing postnatal perineal complications.

## AUTHOR CONTRIBUTIONS

Conceptualization of study was by CB, GAC and LOB. Data collection was performed by CB. Data analysis was performed by GAC. Article drafting was performed by GAC and critically reviewed by CB and LOB.

## CONFLICT OF INTEREST

The authors have no conflicts of interest.

## Data Availability

The data that support the findings of this study are available from the corresponding author upon reasonable request.

## References

[1] Iyengar K , Yadav R , Sen S . Consequences of maternal complications in women's lives in the first postpartum year: a prospective cohort study. J Health Popul Nutr. 2012;30(2):226‐240. doi:10.3329/jhpn.v30i2.11318 22838164 PMC3397333

[2] Jahani Shoorab N , Mirteimouri M , Taghipour A , Latifnejad RR . Women's experiences of emotional recovery from childbirth‐related perineal trauma: a qualitative content analysis. Int J Community Based Nurs Midwifery. 2019;7(3):181‐191. doi:10.30476/IJCBNM.2019.44993 31341917 PMC6614353

[3] Jones K , Webb S , Manresa M , Hodgetts‐Morton V , Morris RK . The incidence of wound infection and dehiscence following childbirth‐related perineal trauma: a systematic review of the evidence. Eur J Obstet Gynecol Reprod Biol. 2019;240:1‐8. doi:10.1016/j.ejogrb.2019.05.038 31202973

